# Role of Evolutionary Selection Acting on Vaccine Antigens in the Re-Emergence of *Bordetella Pertussis*

**DOI:** 10.3390/diseases7020035

**Published:** 2019-04-16

**Authors:** Haley Etskovitz, Nicole Anastasio, Evangeline Green, Meghan May

**Affiliations:** Department of Biomedical Sciences, University of New England, 11 Hills Beach Road, Biddeford, ME 04005, USA; hetskovitz@une.edu (H.E.); nanastasio@une.edu (N.A.); egreen3@une.edu (E.G.)

**Keywords:** Bordetella pertussis, pertussis, whooping cough, vaccine escape, re-emerging disease, pertactin, pertussis toxin, FH

## Abstract

Pertussis (“whooping cough”) is a re-emerging disease with increasing incidence among fully vaccinated individuals. We explored the genetic diversity of five *Bordetella pertussis* proteins used to generate the subunit vaccine across ancestral and newly emergent strains using immunoinformatics and evolutionary selection measurements. The five subunits of pertussis toxin (Ptx1–Ptx5) were highly conserved with regard to sequence, predicted structure, predicted antigenicity, and were under purifying selection. In contrast, the adhesin proteins pertactin (Prn) and filamentous hemagglutinin (FHA) were under statistically significant (*p* < 0.01) diversifying selection. Most heavily diversified sites of each protein fell within antigenic epitopes, and the functional adhesin motifs were conserved. Protein secondary structure was conserved despite sequence diversity for FHA but was changeable in Prn. These findings suggest that subunit vaccine-derived immunity does not impact Ptx1–Ptx5 but may apply evolutionary pressure to Prn and FHA to undergo diversifying selection. These findings offer further insight into the emergence of vaccine-resistant strains of *B. pertussis*.

## 1. Introduction

*Bordetella pertussis* is a human pathogen that causes respiratory illness across all age groups. Infants and children with pertussis experience an acute disease featuring a characteristic hacking, paroxysmal cough [1]. The illness is more extensive in infants and can lead to serious complications, including failure to thrive, apnea, cyanosis, pneumonia, respiratory failure, seizures, and death [2,3]. Infection in adolescents and adults may result in a prolonged cough and is occasionally associated with substantial morbidity [4]. In all age groups, the disease can feature fever and posttussive vomiting [1,4]. Mortality from pertussis most frequently occurs in infants and children, most notably those who are not vaccinated.

**Figure 1:**
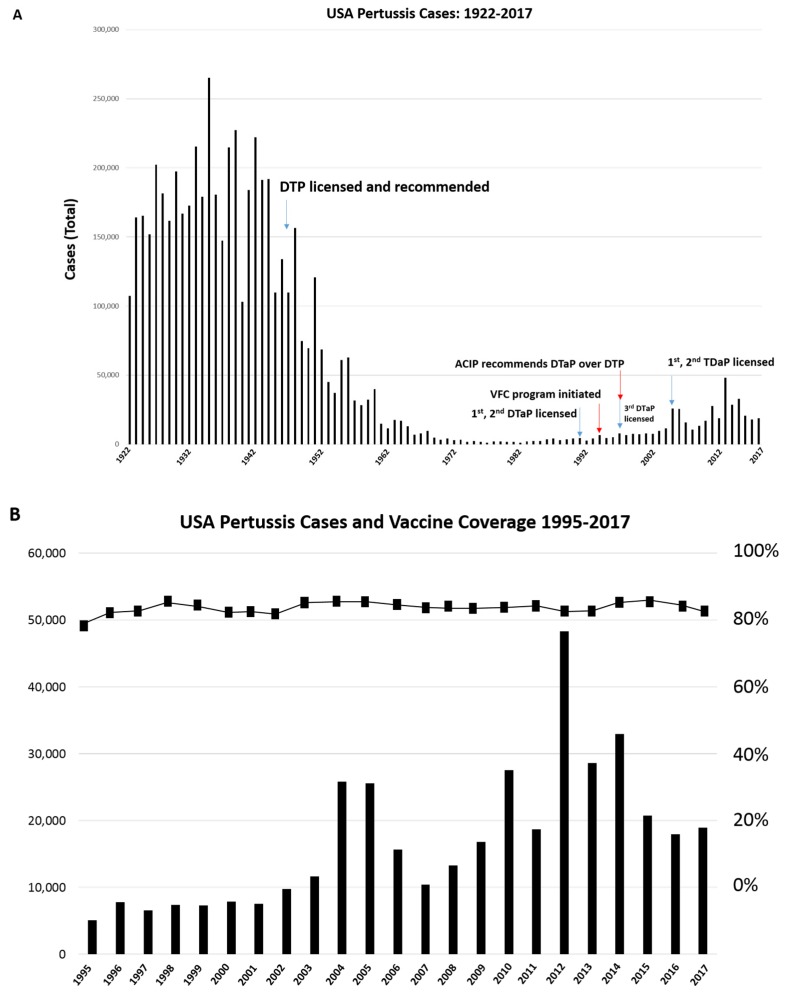
Pertussis cases and vaccine coverage in the United States. (**A**) The total cases of pertussis are depicted between 1922 and 2017. Notable events are marked, including vaccine introductions (blue arrows) and policy measures (red arrows). (**B**) Case numbers (Y axis) between 1995 and 2017 (X axis) have begun to increase despite the relatively stable national vaccine coverage rate (Z axis).

The incidence of pertussis in the United States dropped dramatically following the introduction and widespread recommendation of the diphtheria-pertussis-tetanus (DTP) vaccine in 1947 (Figure 1A), and this trend was mirrored globally. The DTP vaccine was composed of inactivated diphtheria, tetanus, and pertussis toxins, and whole-killed *B. pertussis* cells. The side-effective profile of the DTP vaccine, which included fever and injection site soreness [5], stimulated the development of ‘acellular’ pertussis vaccines, or DTaPs. DTaP vaccines contain multiple purified protein subunits including the adhesins pertactin (Prn), filamentous hemagglutinin (FHA), and, in some formulations, fimbrial antigens FIM2 and FIM3 in addition to the inactivated toxins. The first DTaP vaccines were licensed in 1991, covered for all families in 1995, and formally recommended over DTP vaccines for all children by the Advisory Committee on Immunization Practices (ACIP) in 1997 [6]. An alternative formulation (TDaP) was approved for adults in 2005 (Figure 1A). 

Recently, pertussis incidence in the United States has been increasing [7] despite a relatively stable national immunization rate between 1995 and 2017 (Figure 1B). In addition, cases of pertussis have been diagnosed in fully vaccinated children with alarming frequency, suggesting the emergence of one or more vaccine-refractory strains of *B. pertussis*. These trends have been mirrored worldwide. We sought to analyze this possibility by determining nucleotide and protein sequence diversity of the vaccine antigens from numerous strains collected before and after the introduction of acellular pertussis vaccines; we used these findings to measure points of evolutionary selection and changes within predicted antigenic epitopes. 

## 2. Materials and Methods

### 2.1. Dataset Construction

Pertussis case numbers and vaccination rate data were obtained from the Centers for Disease Control and Prevention via the Pertussis Surveillance and Reporting page (https://www.cdc.gov/pertussis/surv-reporting/cases-by-year.html) and the InstantAtlas™ (NDMSIA) server (https://ndmsia.cdc.gov/IAS/data/excel?viewId=2112&geoId=17&subsetId=&viewer=Excel). Vaccination coverage data reflect the United States national rate for children 19–35 months who received four doses. All nucleotide and protein sequences were mined from GenBank [8]. Strains collected prior to and following the introduction of DTaP vaccines in the country of strain origin were included in this analysis. Strain information (i.e., date of collection, country of origin, local vaccine type available) and accession numbers are available in Supplemental Tables S1–S7.

### 2.2. Evolutionary Selection Analysis 

Sequence evolution occurring in response to diversifying, neutral, or purifying selection was detected using the Selecton v2.4 software suite [9]. Multiple sequence alignments (MSA) exceeding the requisite minimum threshold of 10 sequences were generated for each vaccine component [Ptx1 N = 26 strains; Ptx2 N = 22 strains; Ptx3 N = 30 strains; Ptx4 N = 25 strains; Ptx5 N = 19 strains; Prn N = 52 strains; FHA N = 14 strains]. MSAs were individually interrogated using the M8 model [10,11] to determine selection values as weighted *Ka/Ks* ratios (ω). Sites with ω values of <1 reflect purifying selection, and sites with ω values of ≥1 reflect either neutral (ω = 1) or diversifying (ω > 1) selection. Accordingly, site-specific and global inferences about the forms of selection acting on each protein could be made. The mechanistic model M8 uses maximum likelihood methods where scoring is weighted by different probabilities for transitions and transversions, codon bias, and among-site rate variation to estimate the proportion of codons with ω values of <1 (the beta distribution *p*_0_) and the proportion with ω values of ≥1 (ω*s*). 

### 2.3. Sequence, Immunoinformatic and Structural Analysis 

MSAs with the goal to visualize site-specific amino acid changes were generated using Clustal omega [12]. Predictions of antigenic epitopes were made for Prn and FHA sequences using the parent vaccine strain Tohama using two distinct methods. Probable linear B-cell epitopes were identified using BepiPred v. 2.0 [13], which employs a Random Forest algorithm trained on known epitopes derived from crystal structures. Kolaskar and Tongaonkar residue antigenicity scales were also used to identify potential epitopes by making predictions using the physiochemical properties of protein sequences in comparison to known antigenic sites [14]. Both methods were utilized via the Antibody Epitope Prediction suite of the Immune Epitope Database and Analysis Resource (National Institute for Allergy and Infectious Diseases; www.iedb.org) [15]. Secondary structural predictions were made for Prn and FHA using GOR4 [16]. All unique sequences were modeled; however, multiple strains with identical sequences were only modeled once. Neighbor-joining trees were constructed using Clustal omega [12].

### 2.4. Statistical Procedures

The statistical significance of global selection on each protein for the M8 model calculations was determined via comparison to a null model, M8a. The M8a model simulated neutral or stabilizing selection based on negative deviations from a fixed ω*s* value of 1. Statistical significance was determined by likelihood ratio test between the M8 and M8a model scores. Statistical analyses were carried out using Selecton v2.4 [9]. 

## 3. Results

### 3.1. Evolutionary Selection 

**Figure 2:**
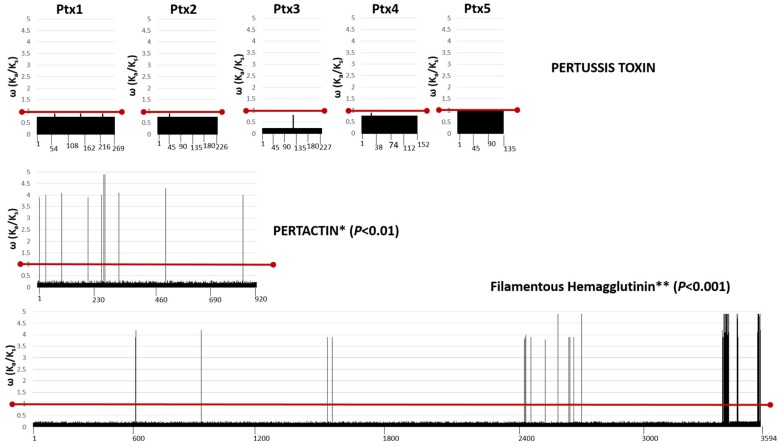
Evolutionary selection acting on vaccine antigens of *B. pertussis*. Amino acid positions (X axis) of all subunits of pertussis toxin (Ptx1–Ptx5; top panel) show evidence of purifying or neutral selection (Y axis; ω ≤ 1, red line). In contrast, both Prn (center panel) and FHA (bottom panel) have numerous amino acid positions under diversifying selection (Y axis; ω ≥ 1, red line), and global rates of diversifying selection acting on the entire protein were significant (*/**, as indicated). Scores derived using the M8 model are shown.

All subunits of pertussis toxin (Ptx1–Ptx5) were under global purifying or neutral selection (Figure 2), with no single amino acid position having a ω value greater than 1. In contrast, both Prn and FHA had multiple sites under diversifying selection (Figure 2 and Figure S1) using either model, indicating global positive selection acts on both proteins. The degree of diversifying selection was statistically significant for both Prn (*p* < 0.01) and FHA (*p* < 0.001). Phylogenetic relationships between strains based on each distinct vaccine antigen are visualized by neighbor-joining trees (Supplemental Figure S1A–F).

### 3.2. Immunoinformatics 

**Figure 3:**
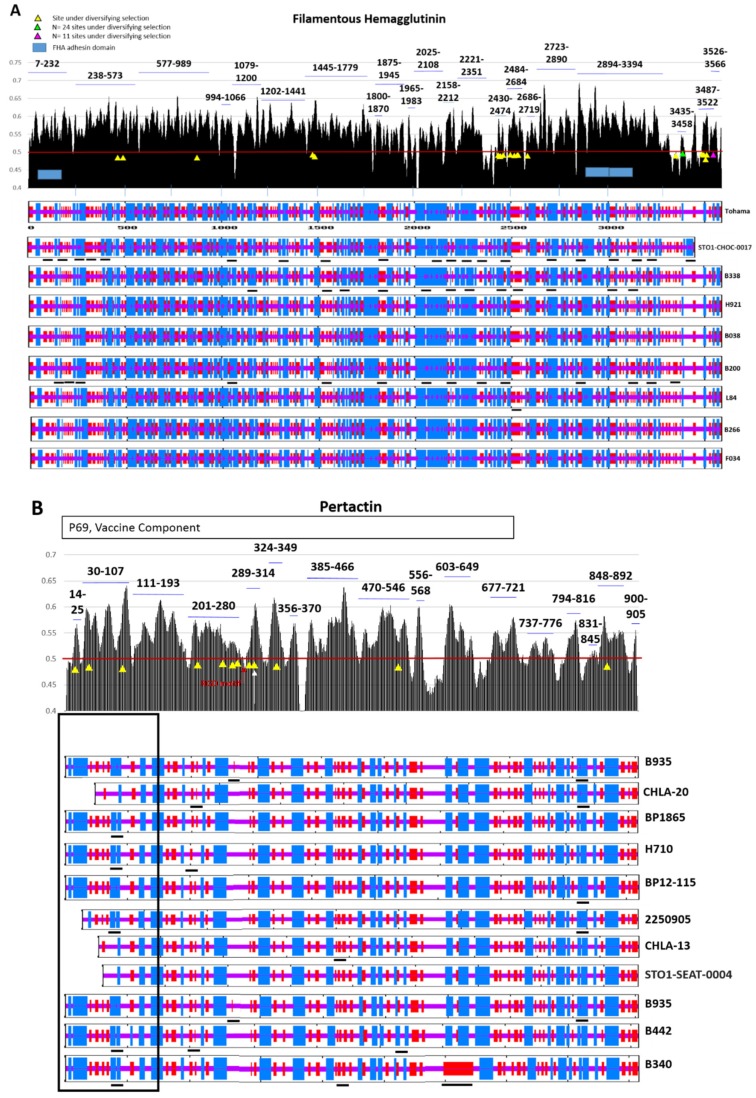
Antigenicity and predicted structure of FHA and Prn. Top panel (**A**,**B**): predicted antigenic epitopes in are depicted graphically by plotting the antigenicity score (Y axis) for each amino acid position (X axis) (blue lines; numbers represent predicted epitopes by amino acid positions). The threshold value of 0.5 for epitope prediction is represented by a red line. Single sites under diversifying selection are shown as yellow triangles. Lower panel (**A**,**B**): Secondary structures for strains representing each unique sequence in the analysis were predicted, and structural prediction diagrams numerically align with the antigenicity plots. Vaccine strain Tohama is depicted and numbered at the top. Alpha helices are depicted as vertical blue bars, coils are depicted as horizontal purple bars, and disordered regions are shown as vertical red bars. Differences in predictions for individuals strains relative to Tohama are underlined in black. (**A**) The functional adhesin domain of FHA is shown as a blue box, and clustered sites under diversifying selection are additionally shown as green (24 sites) or pink (11 sites) triangles. (**B**) The P69 portion of Prn used as an acellular vaccine component is indicated at the top. The *N*-terminal portion containing truncations in multiple strains is boxed, and the RGD adhesion motif of Prn is marked in red. The tandem G-G-(A/G/F)-(V/G)-P repeat region is indicated in the top panel as a white arrow.

Predictions of either linear epitopes (i.e., BepiPred outputs) or epitopes based on physiochemistry (Kolaskar and Tongaonkar analysis; not shown) both indicate that Prn and FHA are highly antigenic proteins. All sites under diversifying selection in Prn and FHA fell within antigenic epitopes predicted by both methods (Figure 3A–B). The functional motifs of both proteins also fell within antigenic epitopes. Antigenicity plots using both methods were similar.

### 3.3. Protein Structure 

**Figure 4:**
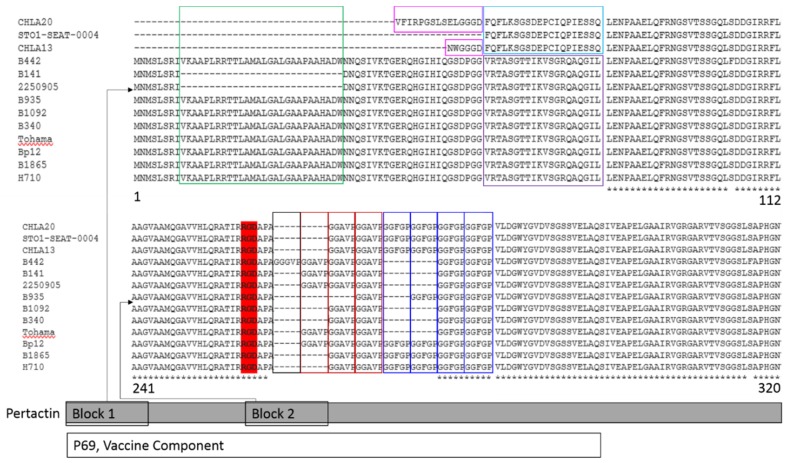
Sequence deletions in pertactin. Specific deletions within Prn are present in multiple strains and fall within two groups. *N*-terminal deletions are shown as “Block 1” (top panel). Three strains examined (CHLA20, CHLA13, and ST)1-SEAT-004) show pure truncations, whereas B141 and 2250905 have an identical 38-residue internal deletion (green box). All strains show homology by position 82, but the upstream sequence for the truncated Prn proteins of strains CHLA20, CHLA13, and STO1-SEAT-004 is unique (pink, light blue boxes) relative to consensus (purple box). Additional insertions/deletions within Prn occur within the tandem G-G-(A/G/F)-(V/G)-P repeat region (“block 2”, lower panel), immediately following the RGD adhesin motif (red highlight). All strains have at least one GGAVP repeat and at least two GGFGP repeats, but additional numbers of GGGVP (black box), GGAVP (red boxes), and GGFGP (blue boxes) vary across strains. Both Blocks 1 and 2 fall within the P69 subunit vaccine component.

Secondary protein structure predictions of Prn and FHA across strains indicates that many, but not all, of the sites under diversifying selection correspond with slight changes in structure (Figure 3A–B). Conversely, some of the significant changes in Prn for individual strains did not appear to have resulted from the diversifying selection as detected by these methods. The carboxyterminal truncation of FHA in strain STO1-CHOC-0017 was immediately preceded by a unique helical stretch, but other strains were structurally consistent in this region (Figure 3A). Aminoterminal truncations or deletions of Prn were observed in multiple strains (Figure 4), inducing structural changes beyond the simple absence of amino acids in these strains (Figure 3B). Structures regained consistency across strains by position 150 with the exception of the length of the coiled-coil motif, which was generated by a diversity in the number of G-G-(A/G/F)-(V/G)-P repeats (Figure 4) and the replacement of a long helical structure with a disordered region at position 600 in strain B340 (Figure 3B).

## 4. Discussion

The increasing incidence of pertussis in fully vaccinated children is alarming. Initial reports indicated that waning immunity is responsible for this increase [17,18]; however, the diversity of circulating isolates warrants reexamination of this conclusion [19,20,21,22,23]. We sought to evaluate diversity in DTaP vaccine antigens through the lens of evolutionary biology, immunoinformatics, and protein structure. The protein sequences of the five pertussis toxin subunits were largely conserved across strains, suggesting that there is no evolutionary pressure to diversify acting on the toxin. In contrast, a statistically significant diversify selection is acting on Prn and FHA, indicating that *B. pertussis* benefits from changing such proteins in response to evolutionary pressure (Figure 2). Phylogenetic relationships between strains based on each distinct vaccine antigen vary by trait, which is consistent with diversity stemming from external pressures rather than inheritance by descent (Supplemental Figure S1A–F).

Ptx, Prn, and FHA are all highly antigenic proteins, and individuals immunized with DTaP or TDaP vaccines raise robust antibody responses against them. All sites under diversifying selection in Prn and FHA occured within predicted antigenic epitopes; all but one site under diversifying selection in Prn occurred within the portion that constitutes the vaccine antigen (P69). The functional domains that mediate attachment of *B. pertussis* to host cells during infection, although antigenic, are themselves under purifying selection (Figure 3B). This presumably reflects the critical role that attachment plays in the disease process as well as in the survival of *B. pertussis* in the human host. Antibody neutralization of the adhesins FHA or Prn would dramatically impact the ability of *B. pertussis* to colonize and survive in the human respiratory tract, creating selective pressure to increase fitness by escaping the raised antibody response.

There are multiple ways to escape an antibody response. The antigenic protein can experience substitution of dissimilar amino acids or deletion of epitopes that compromise antibody avidity while maintaining protein function. Alternatively, if an antigenic protein’s function is redundant or somehow reduced in fitness, the protein can be lost in certain strains. Our analysis shows multiple instances of dissimilar substitutions and deletions within antigenic sites (Figure 3A–B and Figure 4). Numerous Prn-deficient strains capable of causing disease in vaccinated individuals have recently emerged [24,25,26,27,28], and most pertussis isolates from the United States in the past two years are Prn-deficient [29,30]. These strains maintain the capacity for enough adherence to cause disease via FHA, and their decreased adherence appears to confer enhanced fitness over Prn-producing strains in vaccinated individuals within in vivo models of disease [31,32]. Although enhanced fitness suggests that Prn-deficient strains may have overtaken Prn-producing strains in the population, additional selective pressure from neutralizing antibodies during the process of colonization could be accelerating the emergence of these strains. The observed aminoterminal truncations across diverse strains suggests that the partial or full loss of Prn is a favorable trait for *B. pertussis* in populations that are largely DTaP-immunized.

Previous descriptions of Prn diversity have highlighted the expansion or contraction of a tract of tandem G-G-(A/G/F)-(V/G)-P repeats (Figure 4) [33]. Although this does not introduce new predicted structures, it does shorten or lengthen the size of a large coiled-coil motif that immediately follows the function domain (Figure 3B). Bacterial proteins often use coiled-coils as spacers that can alter structure or function [34]. Although the Prn functional motif was conserved across strains, the expansion or contraction of these repeats affected the binding avidity of *B. pertussis* to host cells, allowing a reduction of antibody avidity. FHA similarly exhibited diversifying selection in highly antigenic sites and conservation within its functional domain but did not exhibit the same widespread level of diversity in protein size or presence (Figure 3B). As long as FHA is present and functional, Prn is dispensable. If diversified sites allow for anti-FHA antibody escape without impacting cytoadherence, this might enhance *B. pertussis* fitness. Neutralizing antibodies raised against FHA and, to a lesser extent, Prn would therefore be predicted to exert a strong selective pressure on *B. pertussis* to change the non-functional portions of the proteins.

Our findings of neutral or purifying selection acting on the pertussis toxin subunits was somewhat unexpected. Prior reports describe single nucleotide polymorphism (SNP)-level diversity in Ptx1 [20,33], including at least some substitutions in T-cell epitopes. However, many of the SNPs observed caused substitutions of similar amino acids whose impact on the function, structure, and potential for antibody neutralization were likely minimal. Gzyl et al. reported that, although SNPs could be detected in Ptx1 of circulating strains, most of the variants could be found in at least one vaccine preparation used in one country [20]. SNP-level diversity in the toxin subunits is unlikely to arise from vaccine pressures quickly because the toxin does not play a central role in colonization and bacterial survival, which is where the impact of selection occurs most acutely. Conversely, changes in expression level that lead to enhanced disease can be favorably selected because an increase in severe clinical signs can enhance pathogen transmission [35]. This appears to be the case for the emergent PtxP3 variants, which contain a mutation in their promoters leading to greater toxin gene expression levels and a larger amount of toxin produced [36]. PtxP3 variants spread quickly and cause enhanced disease in vaccinated and unvaccinated populations despite minimal sequence diversity in the Ptx coding sequences [36,37,38,39,40]. The lack of substantial diversity and demonstration of purifying selection indicates that the spread and persistence of PtxP3 variants in vaccinated populations is not due to the escape of anti-Ptx antibodies but results from another mechanism such as Ptx-mediated immune suppression, pressure past the immunological breaking point, or both [41,42].

Immunization rates against pertussis in the United States remained relatively stable between 1995 and 2017 (Figure 1B); however, national rates can mask local trends. Numerous studies have indicated that *B. pertussis* genetic diversity increases in circulating strains after the introduction of the DTP or the DTaP in a country [21,23,43]. The impact between the rate of change following whole cell versus acellular pertussis vaccines is unclear. We illustrated that the most heavily selected sites in the vaccine antigens Prn and FHA fall within antigenic epitopes, which is strongly suggestive of immune selection. This is consistent with many epidemiological patterns and in vivo observations generated in animal models. Strains lacking Prn are isolated twice as frequently from vaccinated children as unvaccinated children [44], and such strains outcompete Prn-producing strains in vaccinated, but not unvaccinated, mice [31,32]. This suggests that the loss of Prn is only favorable when a directed immune response is present. The recent emergence of strains lacking Prn that also feature the PtxP3 allele were initially alarming, but infected patients who had been vaccinated still experienced less severe forms of the disease than those who had not been vaccinated [39].

## 5. Conclusions

The findings presented in this study indicate that the DTaP vaccine is setting the stage for immune selection that will drive diversity in the colonizing factors of *B. pertussis*. Reduction in disease severity among vaccinated individuals is still apparent and the overall risk of pertussis is positively correlated with under-vaccination, indicating that vaccination against pertussis remains a critical public health measure [39,45,46]. Alterations to the current pertussis vaccination strategy are the subject of numerous initiatives [47,48,49,50]. Our findings suggest that further diversification is predictable, and that changes to the current approach are warranted and should be prioritized.

## Supplementary Materials

The following are available online at https://www.mdpi.com/2079-9721/7/2/35/s1, Figure S1: phylogeny of subunit vaccine antigens; Supplemental Tables including strain information, sequence accession numbers, phylogenetic trees, and MSAs. Table S1: Ptx1 strain information, Table S2: Ptx2 strain information, Table S3: Ptx3 strain information, Table S4: Ptx4 strain information, Table S5: Ptx5 strain information, Table S6: Prn strain information, Table S7: FHA strain information. They can be accessed at the following DOIs: Strain Information Tables: DOI: 10.13140/RG.2.2.10572.87682, DOI: 10.13140/RG.2.2.13928.32008, DOI: 10.13140/RG.2.2.34060.97926, DOI: 10.13140/RG.2.2.25672.37121, DOI: 10.13140/RG.2.2.18961.48487, DOI: 10.13140/RG.2.2.32383.25767, DOI: 10.13140/RG.2.2.15606.04167; FHA MSA: DOI: 10.13140/RG.2.2.25865.90722; Prn MSA: DOI: 10.13140/RG.2.2.32576.79368; Phylogenetic Trees: DOI: 10.13140/RG.2.2.29971.53280
