# Supplementary material for: Role of Evolutionary Selection Acting on Vaccine Antigens in the Re-Emergence of Bordetella Pertussis
Source: Diseases. 2019 Apr 16;7(2):35. doi: 10.3390/diseases7020035 (PMC6630436; doi:10.3390/diseases7020035)
Supplement: Supplementary file 1 [file diseases-07-00035-s001.pdf]

A Ptx1

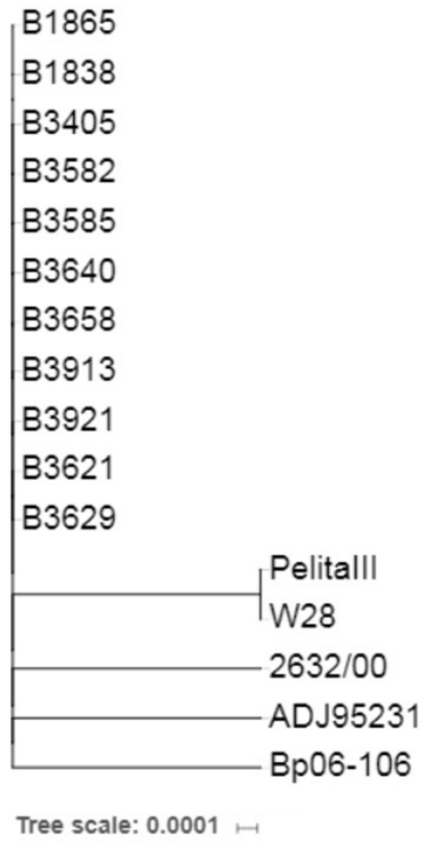
B1865
B1838
B3405
B3582
B3585
B3640
B3658
B3913
B3921
B3621
B3629
PelitaIII
W28
2632/00
ADJ95231
Bp06-106
Tree scale: 0.0001

B Ptx2

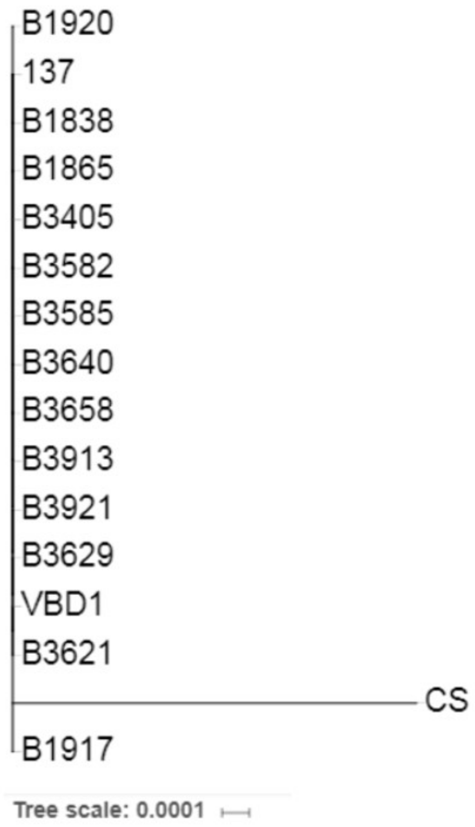
B1920
137
B1838
B1865
B3405
B3582
B3585
B3640
B3658
B3913
B3921
B3629
VBD1
B3621
CS
B1917
Tree scale: 0.0001

C Ptx3

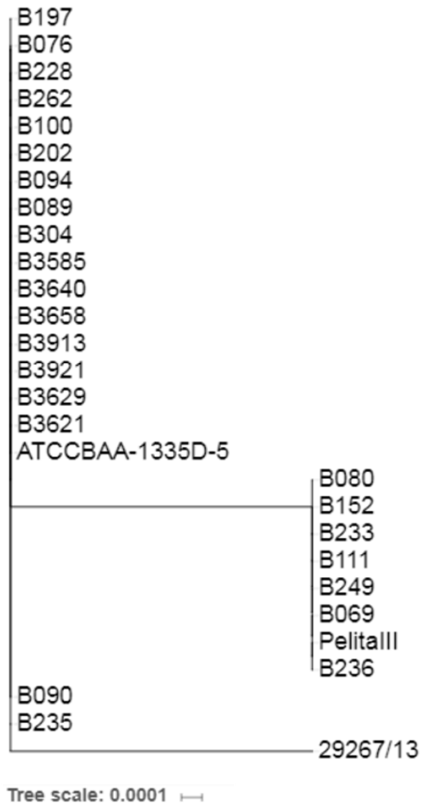
B197
B076
B228
B262
B100
B202
B094
B089
B304
B3585
B3640
B3658
B3913
B3921
B3629
B3621
ATCCBAA-1335D-5
B080
B152
B233
B111
B249
B069
PelitaIII
B236
B090
B235
29267/13
Tree scale: 0.0001

D Ptx4

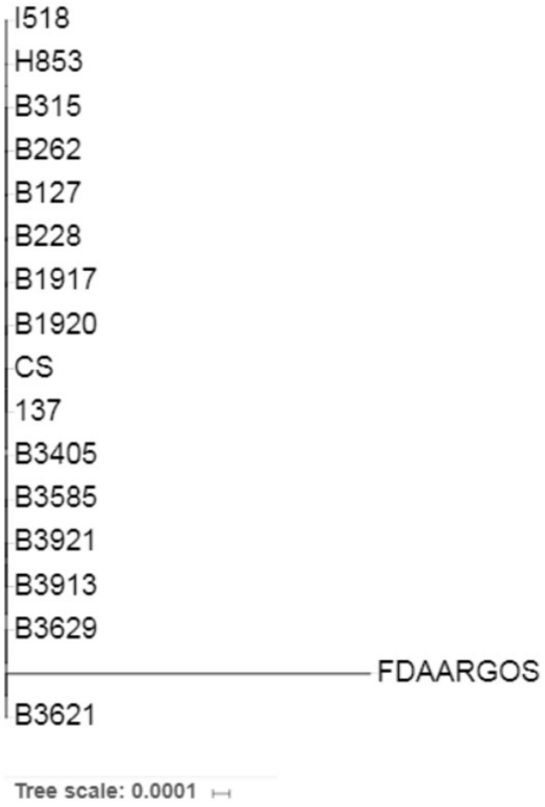
I518
H853
B315
B262
B127
B228
B1917
B1920
CS
137
B3405
B3585
B3921
B3913
B3629
FDAARGOS
B3621
Tree scale: 0.0001

E PRN

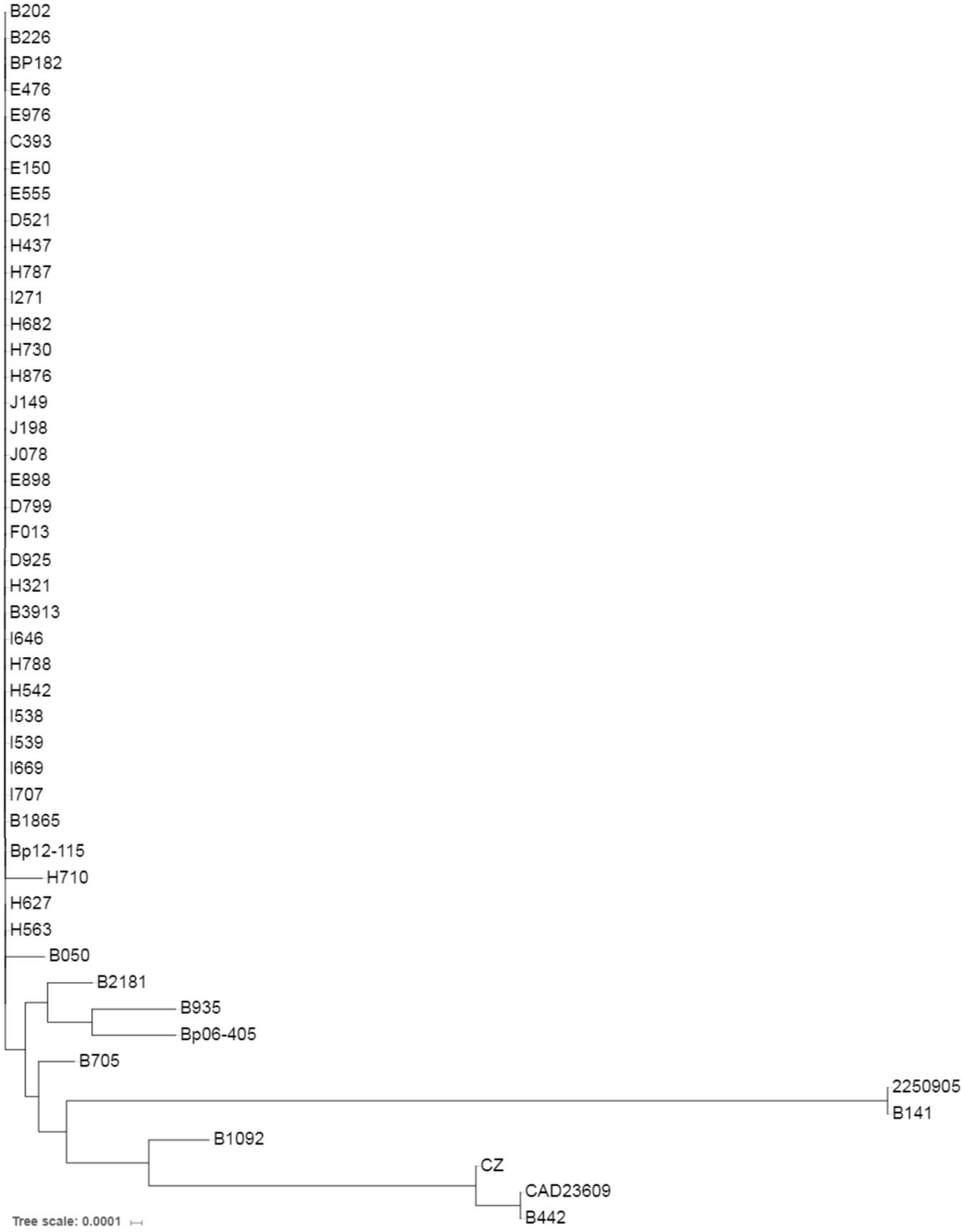
B202
B226
BP182
E476
E976
C393
E150
E555
D521
H437
H787
I271
H682
H730
H876
J149
J198
J078
E898
D799
F013
D925
H321
B3913
I646
H788
H542
I538
I539
I669
I707
B1865
Bp12-115
H710
H627
H563
B050
B2181
B935
Bp06-405
B705
2250905
B141
B1092
CZ
CAD23609
B442
Tree scale: 0.0001

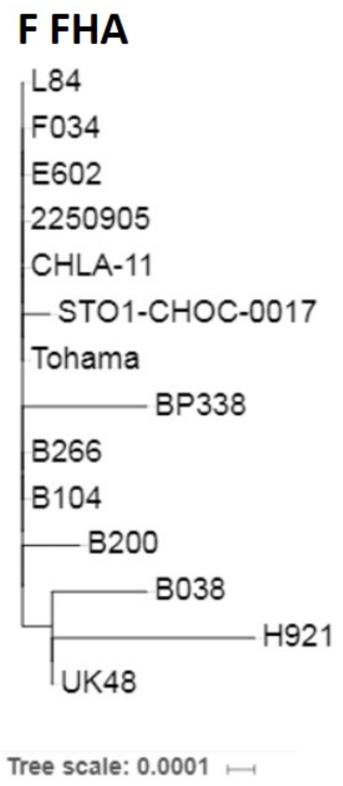

Figure 1. Phylogeny of Subunit Vaccine Antigens. Neighbor-joining trees show the relationships between strains when based on *ptx1* (A), *ptx2* (B), *ptx3* (C), *ptx4* (D), *prn* (E) and *fha* (F). The majority of strains showed minimal to no diversity for *ptx2* and *ptx4*, though there was a single divergent strain for each. While *ptx3* also had a unique divergent strain, it also had two groups of largely homologous strains that fell into two clades. Moderate levels of strains showed divergence in *ptx1* and *fha*. The feature creating the most divergent relationship between strains was *prn*.

Table S1: Ptx1 Strain Information

Accession #	Associated Publications (PMID)	Strain	Isolation Site	Collection Year (if reported)	Introduction Year (wP)	Introduction Year (aP)
AQW66429	28450511	Pelita III	Japan		1948	1981
AHL44849		2632/00	Poland	2000	1960	2004
CAA06893	10418915	18323	USA	1947	1947	1992
ETH90427	24356839	STO1-CHOC-0019	USA	2010	1947	1992

ADA85124		BP103	China		1961	1995
AFP48369		Bp06-106	Canada		1943	1997
AAW72734		CS	China		1961	1995
CAA34397		10536			[redacted]	
CAB51542	9453625	B592	Netherlands		1953	2001
CAA06897	10418915	Fr287	France	1996	1959	1998
CPH55031		B157	Kenya	1975	1970(?)	N/A
ACI04548		BP18	China		1961	1995
ADA85123		BP102	China		1961	1995
ALI23783	26607899	B3621	France	2008	1959	1998
ALI20329	26607899	B3629	France	2009	1959	1998
ALH78728	26607899	B3921	Netherlands	2012	1953	2001
ALH75265	26607899	B3913	Netherlands	2012	1953	2001
ALH71803	26607899	B3658	Norway	2009	1952	1998
ALH68345	26607899	B3640	Netherlands	2010	1953	2001
ALH64880	26607899	B3585	Sweden	2009	1952	1996
ALH61419	26607899	B3582	Sweden	2009	1952	1996
ALH57960	26607899	B3405	Netherlands	2010	1953	2001
ALH54496	26607899	B1865	Netherlands	1999	1953	2001
ADJ95231		N/A	South Korea		1954	1989
CAA06895	10418915	W28	UK	<1950	1957	2001
ALH51037	26607899	B1838	Netherlands	1999	1953	2001

Table S2: Ptx2 Strain Information

Accession #	Associated Publications (PMID)	Strain	Isolation Site	Collection Year (if reported)	Introduction Year (wP)	Introduction Year (aP)
PAX95451		VBD1	India: New Delhi	2017	1978	[redacted]
CFN94433		B020	Australia	1982	1953	1999
CFN02694		B007	Argentina	2014	1960	[redacted]
CPI02170		B035	Australia	1998	1953	1999
CPK58377		B268	Taiwan	1998	1954	1996
CFM12510		B127	Japan	1991	1948	1981
ETA62503	24356839	CHLA-11	USA:CA	2010	1947	1992
ALI23784	26607899	B3621	France	2008	1959	1998
ALI20330	26607899	B3629	Fance	2009	1959	1998
ALH78729	26607899	B3921	Netherlands	2012	1953	2001
ALH75266	26607899	B3913	Netherlands	2012	1953	2001
ALH71804	26607899	B3658	Norway	2009	1952	1998
ALH68346	26607899	B3640	Netherlands	2010	1953	2001
ALH64881	26607899	B3585	Sweden	2009	1952	1996
ALH61420	26607899	B3582	Sweden	2009	1952	1996
ALH57961	26607899	B3405	Netherlands	2010	1953	2001
ALH54497	26607899	B1865	Netherlands	1999	1953	2001
ALH51038	26607899	B1838	Netherlands	1999	1953	2001
AJB24984	25700409	BP137	Brazil		1968	[redacted]
AIW97445	21070624, 25540342	B1920	Netherlands	2000	1953	2001
AIW93985	21070624, 25540342	B1917	Netherlands	2000	1953	2001
AEE68897	21622744	CS	China		1961	1995

Table S3: Ptx3 Strain Information

Accession #	Associated Publications (PMID)	Strain	Isolation Site	Collection Year (if reported)	Introduction Year (wP)	Introduction Year (aP)
AQW66433		Pelita III	Japan		1948	1981
AMG23075		ATCC BAA-1335D-5	USA: VA	1999	1947	1992
AHL44850		29267/13	Poland	2013	1960	2004
CPH86719		B267	Taiwan	1998	1954	1996
CFO05635		B271	Taiwan	1999	1954	1996
CFP56752		B067	Canada	2005	1943	1997
ALI23787	26607899	B3621	France	2008	1959	1998
ALI20333	26607899	B3629	France	2009	1959	1998
ALH78732	26607899	B3921	Netherlands	2012	1953	2001
ALH75269	26607899	B3913	Netherlands	2012	1953	2001
ALH71807	26607899	B3658	Norway	2009	1952	1998
ALH68349	26607899	B3640	Netherlands	2010	1953	2001
ALH64884	26607899	B3585	Sweden	2009	1952	1996
CRD56369		B304	United Kingdom	2005	1957	2001
CPO03579		B089	Finland	2003	1952	2003
CPO82033		B236	Russia	2001	1958	[redacted]
CFV78463		B069	China	1957	1961	1995
CFW05521		B249	Sweden	1970	1952	1996
CFW09585		B111	Italy	1994	1967	1992
CFW54376		B094	Finland	2006	1952	2003

CFW87588		B233	Poland	1974	1960	2004
CFU22575		B090	Finland	2003	1952	2003
CFO93719		B202	Netherlands	2000	1953	2001
CFO18372		B100	France	2000	1959	1998
CFN65272		B152	Kenya	1975	1970(?)	[redacted]
CFN10849		B080	Finland	1964	1952	2003
CFM46393		B262	Sweden	2004	1952	1996
CFL80418		B228	Poland	1999	1960	2004
CPK24577		B076	Denmark	2002	1961	1997
CPJ56719		B197	Netherlands	1996	1953	2001
CPO98387		B235	Poland	2000	1960	2004

Table S4: Ptx4 Strain Information

Accession #	Associated Publications (PMID)	Strain	Isolation Site	Collection Year (if reported)	Introduction Year (wP)	Introduction Year (aP)
PNO99246		FDAARGOS_178	USA: VA	1952	1947	1992
CAE44040	12910271	Tohama	Japan	1952	1948	1981
CFN60288		B130	Japan	1995	1948	1981
KCV22360		B200	USA	1935	1947	1992
ETA62552		CHLA-11	USA:CA	2010	1947	1992
CFP40933		B002	Argentina	2000	1960	[redacted]
CPP60490		B066	Canada	2004	1943	1997
CPM23664		B273	Taiwan	2000	1954	1996
CFO05395		B271	Taiwan	1999	1954	1996

ALI23785	26607899	B3621	France	2008	1959	1998
ALI20331	26607899	B3629	France	2009	1959	1998
ALH78730	26607899	B3921	Netherlands	2012	1953	2001
ALH75267	26607899	B3913	Netherlands	2012	1953	2001
ALH64882	26607899	B3585	Sweden	2009	1952	1996
ALH57962	26607899	B3405	Netherlands	2010	1953	2001
AEE68898	21622744	CS	China		1961	1995
AJB24983	25700409	B137	Brazil		1968	[redacted]
AIW97446	21070624, 25540342	B1920	Netherlands	2000	1953	2001
AIW93986	21070624, 25540342	B1917	Netherlands	2000	1953	2001
CFL80422		B228	Poland	1999	1960	2004
CFM12507		B127	Japan	1991	1948	1981
CFM46397		B262	Sweden	2004	1952	1996
CPK71839		B315	USA	1986	1947	1992
CAX52278	19751581	B1831	Netherlands	1999	1953	2001
AQF09999	28167525	H853	USA: MN	2011	1947	1992

Table S5: Ptx5 Strain Information

Acession #	Associated Publications (PMID)	Strain	Isolation Site	Collection Year (if reported)	Introduction Year (wP)	Introduction Year (aP)
AQW66432	28450511	Pelita III	Japan		1948	1981
ETH02262	24356839	2250905	USA		1947	1992
ALI23786	26607899	B3621	France	2008	1959	1998

ALH78731	26607899	B3921	Netherlands	2012	1953	2001
ALH68348	26607899	B3640	Netherlands	2010	1953	2001
AEE68899	21622744	CS	China		1961	1995
AIW97447	21070624, 25540342	B1920	Netherlands	2000	1953	2001
AIW93987	21070624, 25540342	B1917	Netherlands	2000	1953	2001
AJB24982	25700409	BP137	Brazil		1968	[redacted]
AMT67768	27303739, 28167525	I475	USA: VT	2012	1947	1992
ANT88056	27635001, 28167525	B203	USA: MI	1939	1947	1992
AMT47043	27303739, 28167525	I538			[redacted]	
CPK48971		B322	USA	1998	1947	1992
PNO99247		FDAARGOS _178	USA: VA	1952	1947	1992
CFM49876		B143	Kenya	1975	1970(?)	[redacted]
CRD56374		B304	United Kingdom	2005	1957	2001
CPO35473		B018	Australia	1977	1953	1999
AQB50113		F684	USA: NC	2008	1947	1992
AQH20440	28167525	J068	USA:CT	2013	1947	1992

Table S6: Prn Strain Information

Accession #	Associated Publications (PMID)	Strain	Isolation Site	Collection Year (if reported)	Introduction Year (wP, aP)	Size (AA)	Tandem repeats* (GGAVP+GGFGP)
AMS70425	27303739, 28167525	C393	China	1951	1961, 1995	910	3+2
ABO77783		B2181	Sweden		1952, 1996	900	2+1
AGS56996		B050			[redacted]	910	3+2
ALH53583	26607899	B1865	Netherlands	1999	1953, 2001	915	2+4
ALH72819	26607899	B3913	Netherlands	2012	1953, 2001	915	2+4
ALX22215	26812174, 28167525	H321	France	2007	1959, 1998	915	2+4
AMS55851	27303739, 28167525	H542	USA: CA	2010	1947, 1992	915	2+4
AMS63008	27303739, 28167525	H563	USA: CA	2010	1947, 1992	915	2+4
AMS72356	27303739, 28167525	E476	Sweden		1947, 1992	910	3+2
AMS97778	27303739, 28167525	H627	USA: CA	2010	1947, 1992	915	2+4
AMT06552	27303739, 28167525	H788	USA: VT	2011	1947, 1992	915	2+4
AMT44695	27303739, 28167525	I538	USA: VT	2012	1947, 1992	915	2+4
AMT48297	27303739, 28167525	I539	USA: VT	2012	1947, 1992	915	2+4
AMT51897	27303739, 28167525	I646			1947, 1992	915	2+4
AMT60578	27303739, 28167525	I669	VT	2012	1947, 1992	915	2+4
AMT64217	27303739, 28167525	I707	VT	2012	1947, 1992	915	2+4
ANT92518	27635001	B202	USA: PA	1946	1947, 1992	910	3+2
AQB09332	28167525	D521	USA: MN	2000	1947, 1992	915	2+4
AQB16620	28167525	E150	USA: OH	2003	1947, 1992	915	2+4
AQB20215	28167525	E555	USA: MT	2004	1947, 1992	915	2+4

AQB29615	28167525		E976	USA: NY	2005	1947, 1992	910	3+2
AQD30432	28167525		H710	USA: NY		1947, 1992	915	2+4
AQE01189	28167525		H437	USA: TN	2006	1947, 1992	915	2+4
AQE73291	28167525		H787	USA: MN	2011	1947, 1992	915	2+4
AQF77983	28167525		I271	USA: NY	2012	1947, 1992	915	2+4
AQH84349	28167525		H682	USA: PA	2009	1947, 1992	915	2+4
AQH96721	28167525		H730	USA: MA	2011	1947, 1992	915	2+4
AQJ16226	28167525		H876	USA:OR	2012	1947, 1992	915	2+4
AQJ67331	28167525		J149	USA: OR	2014	1947, 1992	915	2+4
AQJ96352			J198	USA: CO	2014	1947, 1992	915	2+4
ARM10248			Bp06-405	Canada		1943, 1997	915	2+4
ARM10249			Bp12-115	Canada		1943, 1997	920	3+4
ASR57979			J078	USA: MN	2013	1947, 1992	915	2+4
ASX33144			B226	United Kingdom	1967	1957, 2001	910	3+2
ASX51355			E898	USA: AZ	2005	1947, 1992	915	2+4
ASX54989			D799	USA: ID	2002	1947, 1992	915	2+4
ASX62256			F013	USA: NE	2005	1947, 1992	915	2+4
ASX74629			D925	USA: NY	2002	1947, 1992	915	2+4
BAF35031		18291563	BP182	Japan: Fukuoka		1948, 1981	915	3+3
CAA06902	10418915		CZ	France	1993	1959, 1998	912	3*+2
CAA09460	10338531		B705	Finland		1952, 2003	905	2+2
CAA09461		10463173	B935	Netherlands	1995	1953, 2001	905	1+3
CAB39891		9453625	B1092	France		1959, 1998	905	2+2

CAD23609		B442	Netherlands		1953, 2001	912	4*+2
CPL11682		B141	Japan	2006	1948, 1981	882	3+2
CPO86300		B340	USA	2004	1947, 1992	905	2+2
ETG98692	24356839	2250905	USA		1947, 1992	882	3+2
ETH14359.1	24356839	STO1-SEAT-0007	USA:	2010	1947, 1992	915	2+4
ETH20458	24356839	CHLA-13	USA	2010	1947, 1992	862	2+4
ETH27544	24356839	CHLA-20	USA	2010	1947, 1992	870	2+4
ETI01411	24356839	STO1-SEAT-0004	USA	2011	1947, 1992	855	2+4
NP_879839	12910271	Tohama I	Japan	1952	1948, 1981	910	3+2

*Indicates at least one GGAVP repeat is GGGVP

Table S7: FHA Strain Information

Accession #	Associated Publications (PMID)	Strain	Isolation Site	Collection Year (if reported)	Introduction Year (wP)	Introduction Year (aP)	Size (AA)
CAE42162	12910271	Tohama	Japan	1952	1981	1948	3590
ETA65616		CHLA-11	USA	2010	1992	1947	3590
ETG99609		2250905	USA		1992	1947	3590
SUV74174		L84	UK	1950	2001	1957	3590
CPM47677		B266	Taiwan	1997	1996	1954	3590
CPJ45980		B104	France	2004	1998	1959	3590
AQB37449	28167525	F034	USA (CA)	2005	1992	1947	3590
AQH78048	28167525	E602	USA (DE)	2005	1992	1947	3590
RDV18794		UK48	UK	2012	2001	1957	3590
KCV18222		B200	USA	1935	1992	1947	3590*
ETH82271	24356839	STO1-CHOC-0017	USA	2010	1992	1947	3442**
AAA22974	2539596	BP338	Unknown	Unknown	[redacted]		3591
ETH45731	24356839	H921	USA	2012	1992	1947	3590
CFU21572		B038	Australia	2000	1999	1953	3590

*Upon viewing the submitted contig (Bordetella pertussis B200 ctg000033), it is clear that the start site is called incorrectly. There is no truncation rrelative to other strains (entry reports length is 3530). The the full-length sequence is available in the contig (Accession JGWE01000166 https://www.ncbi.nlm.nih.gov/nuccore/627859027

**Middle of the contig - CTD truncation likely real and not assembly error
